# Association of cerebellar volume with cognitive and motor function in adults with congenital heart disease

**DOI:** 10.1007/s10072-023-06861-2

**Published:** 2023-06-23

**Authors:** Nadja Naef, Selma J. Hottinger, Ladina Schlosser, Matthias Greutmann, Beatrice Latal, Ruth Tuura O’Gorman

**Affiliations:** 1 Child Development Center, University Children’s Hospital Zurich, Zurich, CH Switzerland; 2 Department of Neurology, University Hospital Zurich, Zurich, Switzerland; 3 Department of Cardiology, University Heart Center, University Hospital Zurich, Zurich, Switzerland; 4 Children’s Research Center, University Children’s Hospital Zurich, Zurich, CH Switzerland; 5 MR Research Center, University Children’s Hospital Zurich, Zurich, CH Switzerland

**Keywords:** Congenital heart disease, Executive function, Cerebellum, MRI, Brain imaging, Adult

## Abstract

**Introduction:**

Patients with congenital heart disease (CHD) are at risk for cognitive and motor function impairments, brain injury, and smaller total brain volumes. The specific vulnerability of the cerebellum and its role in cognitive and motor functions in adults with congenital heart disease is not well defined.

**Methods:**

Forty-three patients with CHD and 53 controls between 18 and 32 years underwent brain magnetic resonance imaging and cognitive, executive (EF), and motor function assessment. Cerebellar volumes were obtained using EasyMeasure and SUIT Toolbox. Associations between cerebellar volumes and cognitive and motor function were calculated using linear models.

**Results:**

General cognitive and pure motor functions were lower in patients compared to controls (*P* < 0.05). Executive functions were within the normal range. While total cerebellar volumes and the anterior lobes were similar in patients and controls (*P* > 0.1), the posterior cerebellar lobe was smaller in patients with more complex CHD (P = 0.006). Smaller posterior cerebellar gray matter was not associated with cognitive functions. Smaller anterior cerebellar gray matter was not significantly related to motor functions (*P* > 0.1).

**Conclusion:**

In adults with CHD, cerebellar volume was largely unimpaired. Patients with more complex CHD may be vulnerable to changes in the posterior cerebellar gray matter. We found no significant contribution of cerebellar gray matter to cognitive and motor impairments. More advanced imaging techniques are necessary to clarify the contribution of the cerebellum to cognitive and motor functions.

**Supplementary Information:**

The online version contains supplementary material available at 10.1007/s10072-023-06861-2.

## Introduction

Complex CHD is a significant contributor to birth-defect-related morbidity [1, 2] and is associated with an increased risk of motor and neurocognitive function deficits [3]. In the 3rd trimester, CHD fetuses show a progressive decline in global brain growth, likely resulting from altered fetal circulation leading to abnormal oxygen and nutrient delivery [4, 5]. During this fetal period, the cerebellum is the region with the most rapid growth in healthy fetuses, suggesting a susceptibility of the cerebellum to hypoxic injury due to its high metabolic demand [6, 7]. One study found evidence of cerebral cortex dysmaturation within the microstructure in CHD neonates, where reduced cerebral oxygen delivery was related to impaired dendritic arborization, possibly making the cerebellum a particularly vulnerable structure to volumetric impairment as it is mainly comprised of gray matter [8]. In fact, smaller cerebellar volumes, along with smaller global brain volumes, have been previously observed in neonates, adolescents, and young adults with CHD [9–11]. However, the burden of cerebellar hypoplasia on neurodevelopmental outcomes remains mostly unclear.

It is well recognized that the cerebellum plays an important role in sensorimotor function, which is thought to be mainly associated with the anterior cerebellar lobe [12, 13]. In addition, current evidence indicates that the cerebellum plays a key role in cognitive and behavioral function. In particular, the posterior cerebellar lobe is thought to modulate cognitive processes via subcortical connections to the basal ganglia and the thalamus forming a cerebro-cerebellar loop [14], connections to the prefrontal cortex through the cerebello-thalamo-cortical and cortico-ponto-cerebellar pathways, passing through the superior and middle cerebellar peduncles [15, 16]. Functional magnetic resonance imaging (fMRI) studies describe activity in the posterior cerebellar lobules during cognitive tasks, such as working memory and inhibition, suggesting that the posterior cerebellum is engaged during a variety of executive function (EF) tasks as part of a functional network [14, 17–19].

Analysis of fMRI data from the Human Connectome Project including 787 subjects revealed cognitive task activation of lobule VI and VII, crus I II and lobule VIIB, IX, X of the cerebellum [20]. However, spatial compartmentalization of sensorimotor and cognitive function in the cerebellum may vary among individuals. One study found working memory and language tasks mainly activated regions in the posterior cerebellum (lobule VI and crus I). They found that activation patterns varied betwee individuals [18]. In addition, individual cerebellar areas are likely involved in more than one cognitive task, consistent with the modulating function of the cerebellum [18, 19].

Difficulties resulting from lesions in the posterior lobe are summarized in the cerebellar cognitive, affective syndrome and include executive function, spatial tasks, linguistic skills, and regulation of effect [19, 21]. The association of cognitive tasks with cerebellar volume has been investigated in a variety of studies and is likely age dependent [12, 14, 17]. In typically developing adolescents, higher scores on vocabulary, reading, working memory, and set-shifting were associated with increased gray matter volume in the posterior cerebellar lobe [12]. They found that the strength of the positive structure-function relationship of cerebellar gray matter and cognitive tasks was stronger with increasing age until adolescence. Another study found an association of cerebellar volume with working memory in children, but not in adults [14]. The cerebellum is involved in skills acquisition, which is particularly important during early childhood, and appears to be less important for the retention of learned behaviors [17]. Cerebellar recruitment may thus be more specialized and hence weaker in adulthood as tasks become more automated.

 Preterm-born children comprise another population at risk for neurodevelopmental impairment, likely due to a vulnerability of the immature brain similar to the CHD population [22]. In preterm-born children, cerebellar injury and impaired cerebellar growth are frequently reported and associated with poor motor functions, poor cognitive and language functions, and behavioral problems consistent with the cerebellar cognitive, affective syndrome [23–26]. Smaller cerebellar volumes were associated with worse IQ in prematurely born adolescents. Similarly, an association of smaller cerebellar volume with worse motor performance was found in prematurely born children [23, 24]. Neurodevelopmental impairments in preterm-born and CHD population are similar, suggesting similar underlying structural impairments. In the preterm population, cerebellar volumetric impairment likely contributes to neurocognitive dysfunction. Similarly, the CHD population may be particularly susceptible to cerebellar hypoplasia due to impaired intrauterine growth, further highlighting the importance to study the cerebellum in the CHD population [27].

In CHD adults, information on the contribution of cerebellar volumes to executive impairments is rarely investigated, despite evidence suggesting that EF deficits persist into adulthood [28]. In CHD infants, perioperative cerebellar volume was associated with behavior regulation, fine motor function, and cognitive abilities [29]. Another study in adolescents with CHD found an association of cerebellar volume with a variety of executive function tasks, including working memory, cognitive flexibility, and inhibition [30]. In addition, cerebellar volume modulated the relationship between the prefrontal cortex and executive functioning [30]. One small study in CHD adults reported smaller volumes in the posterior cerebellum associated with poorer EF performance in the color-word-interference task, letter-number sequencing, matrix reasoning, and the BRIEF questionnaire and poorer motor performance in the grooved pegboard task [11]. However, Semmel and colleagues were not able to establish a link between the anterior cerebellum and motor function, measured with a pegboard task [11]. 

Therefore, the purpose of this study was to describe the burden of cerebellar volumetric alterations in the CHD population. Anterior and posterior lobes were analyzed separately to account for the spatial compartmentalization of the cerebellum, where the regions typically engaged during cognitive tasks are mainly located in the posterior cerebellar lobe [11, 12, 14, 17, 18]. We investigated the association of cerebellar gray matter with executive, motor function, processing speed, and the maintenance of attention in adults with CHD in comparison to healthy peers using a broad assessment battery of executive functions. We hypothesize that cerebellar gray matter is smaller in the CHD population and that smaller cerebellar volumes are associated with worse cognitive and motor function.

## Methods

### Study design

In this cross-sectional study, participants underwent neuropsychological assessments at the Zurich University Hospital and magnetic resonance brain imaging (MRI) at the University Children’s Hospital Zurich.

### Study population

Young adults with CHD aged 18 to 32 years were recruited from two previous studies [31] between 2016 and 2018 at the University Hospital Zurich. Exclusion criteria for patients were as follows: genetic disorder, congenital or acquired neurological or psychiatric disorder affecting intellectual development (i.e. severe cerebral palsy, severe depression, or mental illness, preventing the patient from completing the neurocognitive assessment), or if patients were not fluent in the German language. Healthy controls matched to the adults with congenital heart disease (ACHD) were recruited as peers of the ACHD or through personal contacts. Controls were screened for comorbidities by means of a questionnaire and were excluded if they had any neurological or psychiatric condition. Of 67 ACHD, all underwent neuropsychological assessment and 46 underwent brain imaging (21 patients did not undergo MRI for the following reasons: MRI safety not ensured *n* = 12, claustrophobia *n* = 2, obesity *n* = 1, pregnancy *n* = 1, refused *n* = 5). The 21 patients who did not undergo MRI were not significantly different in disease complexity, presence of cyanosis, number of cardio-pulmonary bypass surgeries, age at first bypass surgery, and estimated IQ (*P* > 0.08).

### Demographic variables

Demographic variables were collected through questionnaires: Years of education were measured as the number of school years until the completion of an initial education, which in Switzerland usually consists of 12 years of education for vocational training, 15 years for a bachelor’s degree, and 17 years for a master’s degree. Parental education was estimated based on maternal and paternal educational levels, using a 12-point scale ranging from 2 (lowest) to 12 (highest) parental education.

### Cognitive function

Participants underwent comprehensive neurocognitive assessment, according to a test battery described previously [32].

### IQ

Intelligence quotient (IQ) was estimated using a short form of the Wechsler Adult Intelligence Scale, Fourth Edition (WAIS-IV)[33].

### Executive function

EF was assessed using an extensive test battery to capture the multidimensional and overlapping nature of executive functions [34, 35]. The following tests were included: the color-word-interference (CWI) test of the Delis-Kaplan Executive Function System (D-KEFS) [36] to assess inhibitory control and cognitive flexibility; the 5-Point Test [37] to evaluate nonverbal fluency; the S-word subtest of the Regensburger Wortflüssigkeitstest (RWT) [38] to assess verbal fluency; Standardized Link’s Probe (SLP) to assess goal-oriented action planning and constructive solution behavior [39]; Trail Making Test (TMT) to assess flexibility [40]; the computer-based stop signal task (SST) [41] was applied to measure response inhibition; the subtest verbal digit from the (WAIS-IV) [42] and visual block span (WMS-R) [43] were used to measure working memory. The Rey-Osterrieth Complex Figure (ROCF) was administered to measure visuomotor planning skills. This task requires participants to copy a complex figure on a blank paper as quickly and accurately as possible [44]. Test results were expressed as T-values according to the respective test manuals. From the T-values, an average was calculated to form a mean score for flexibility, inhibition, working memory, planning, visuomotor, and fluency to reduce the number of statistical tests performed. The following variables were used to form average scores: working memory: visual block span of the WMS-R and verbal digit span; inhibition: CWI and SST test; flexibility: CWI and TMT; planning: SLP and ROCF; fluency: 5-Point Test and the S-word subtest (RWT). For further details, see Supplemental Table 1.

### Attention

An attention score was formed from the reaction times to visual and auditory presented stimuli of the Test of Attentional Performance Test [45].

### Processing speed

A processing speed score was formed from the first condition of the color-word test [36], a measure for color processing speed, and the first condition of the trail making test, a measure for cognitive processing speed [40].

### Neuromotor function

Pure and adaptive motor functions were assessed using the Zurich Neuromotor Assessment [46] from the time needed to fulfill the task. Pure motor functions include repetitive and alternating hand and foot movements as well as sequential finger movements. Adaptive motor functions were measured with a pegboard task. Results were expressed as z-scores.

### Brain magnetic resonance imaging

#### Image acquisition

Participants underwent a research cerebral MRI scan on a 3T GE MR750 scanner. Hearing protection was provided with earplugs and headphones. For volumetric analysis, high-resolution three-dimensional (3D) T1-weighted images were acquired using a 3D spoiled gradient echo (SPGR) pulse sequence (TR = 11 ms, TE =5 ms, inversion time = 600 ms, FOV = 256 mm, matrix =256 × 192 mm, ST = 1 mm, flip-angle = 8°).

#### MRI processing

Total brain volume was obtained with FreeSurfer image analysis suite version 5.3.0, which is documented and freely available for download online (http://surfer.nmr.mgh.harvard.edu/). MRI images were then processed with EasyMeasure to obtain total cerebellar volumes and the SUIT toolbox to obtain cerebellar lobal volume. SUIT is a toolbox for the SPM (statistical parametric mapping) software used in Matlab. SUIT is developed to handle high inter-subject variability in the cerebellar anatomy. Preprocessing was done according to SUIT documentation, followed by isolation, normalization, and segmentation in the SUIT macro. Unlike the other segmentation methods, SUIT provides the volume of the individual cerebellar lobes, which can be added together to derive the volume of the cerebellar gray matter, but does not include the cerebellar white matter [11, 47]. To limit the number of statistical tests, we decided to combine lobules into anterior and posterior cerebellar gray matter instead of investigating single lobules. Total cerebellar volumes were obtained using the Cavalieri method of design-based stereology in combination with point counting using the EasyMeasure software. Stereological measurement of the cerebellum requires the determination of the number of test points that intersect the cerebellum using an array of probes overlaid onto the MR image and the slice thickness. For each slice, points are counted for each square grid of test points, overlaid on each image with a new random orientation of the square grid. The volume is computed as the sum of point count per section multiplied by the area covered by each test point and again multiplied by the sectioning interval. The grid size was selected to result in a coefficient of error (CE) below 5% [48]. The segmentation of 10 cases (5 per group) was performed twice by 2 raters (S.H. and N.N.) with an average percentage difference of 1.64% and mean coefficient of variation of 0.03. The remaining cases were segmented by one rater (S.H.).

### Statistics

Descriptive statistics include mean and standard deviation (SD) or median and interquartile range (IQR) and frequencies. Group differences were calculated using the t-test and, for non-normal variables, the Mann-Whitney U test. The χ^2^ test was used for categorical variables. Non-normally distributed variables were log-transformed. Differences in cerebellar volumes between groups were calculated using linear regression, adjusted for total brain volume to account for global brain volumetric differences. The association of cerebellar volume with cognitive and motor functions was analyzed using linear regression with cognitive/motor function as the dependent variable and cerebellar volume as the independent variable. In the patient group only, the influence of the CHD category (simple, moderate, and complex) [49] on cerebellar volumes was analyzed using linear regression, adjusted for total brain volume. For regression models, estimates, standardized beta, 95% confidence interval (CI), and *P*-values of the effect are reported. Two-tailed *P*-values <0.05 were considered significant. In addition, the overall *P*-values of the model of the brain volumetric difference and association of cerebellar volume with cognitive and motor function were adjusted for multiple comparisons with FDR for three analyses separately (comparison of brain volumes, association of posterior cerebellum and cognitive function, association of anterior cerebellum and motor function) [50]. Analysis was conducted with the R software (R core team, version 3.4.2, URL https://www.Rproject.org/.).

## Results

### Study population

The study population was described previously [31]. A total of 46 patients and 54 controls underwent assessment of general cognitive ability, executive and motor function, and brain imaging. MRI processing with the SUIT toolbox and EasyMeasure was successful in 96 subjects. Thus, we report data of 43 ACHD (17 female) and 53 controls (26 female) assessed at a mean age of 26 years. ACHD and controls did not differ in sex and age (*P* > 0.1). Parental education was similar in ACHD and controls (8 (7.5; 10) vs. 9 (8; 10), *P* = 0.10). Median years of education were lower in ACHD than in controls (13 (12; 15) vs. 15 (14; 16), *P* = 0.010). All participants were of the Caucasian race. Cardiac diagnoses are listed in Supplemental Table 2. A total of 30 (69.8%) patients underwent at least one surgery requiring cardio-pulmonary bypass. Of these patients, 10 (21.3%) underwent more than one surgery on cardio-pulmonary bypass. Two subjects had a univentricular heart defect, and 12 (27.9%) had a repaired cyanotic defect. In the patient group, 15 patients had simple, 20 moderate, and 8 complex CHD. The median age at first bypass surgery was 1.38 (0.5; 6.2) years.

Subject outcome variables are presented in Table 1. IQ, attention, processing speed scores, and pure motor function were significantly lower in ACHD compared to controls (*P* < 0.05). However, EF functions were not different between the two groups (*P* > 0.1).

**Table 1:** Subject characteristics and outcome variables

M (SD)	ACHD, *N* = 43	Controls, *N* = 53	Effect size (Cohen’s *d*)	*P*
IQ Mild impairment, *N* (%)	97.5 (10.77) 6 (14.0)	104.02 (12.15) 1 (1.9)	−0.56	0.008 0.039
Inhibition Mild impairment, *N* (%)	48.87 (4.63) 2 (4.7)	49.56 (6.88) 1 (1.9)	−0.11	0.578 0.854
Working memory Mild impairment, *N* (%)	50.42 (6.21) 3 (7.0)	52.83 (6.63) 2 (3.8)	−0.37	0.071 0.810
Flexibility Mild impairment, *N* (%)	50.74 (6.50) 4 (9.3)	50.89 (5.58) 4 (7.5)	−0.02	0.908 1.000
Fluency Mild impairment, *N* (%)	50.37 (6.58) 6 (14.0)	52.61 (5.21) 0 (0)	−0.38	0.066 0.017
Planning Mild impairment, *N* (%)	46.55 (9.25) 12 (27.9)	48.44 (7.98) 5 (9.4)	−0.22	0.290 0.059
Attention Mild impairment, *N* (%)	44.36 (6.15) 13 (30.2)	47.52 (7.04) 9 (17.0)	−0.47	0.023 0.196
Processing speed Mild impairment, *N* (%)	50.86 (6.06) 4 (9.3)	53.33 (5.88) 1 (1.9)	−0.41	0.046 0.244
Pure motor function Mild impairment, *N* (%)	−0.38 (1.12) 12 (27.9)	0.41 (1.22) 6 (11.3)	−0.67	0.002 0.071
Adaptive motor function Mild impairment, *N* (%)	0.45 (0.90) 1 (2.3)	0.32 (1.12) 5 (9.4)	0.13	0.532 0.314

*IQ*, intelligence quotient; *M*, mean; *SD*, standard deviation

Mild impairment defined as a performance score below −1 SD (IQ below 85, T-value below 40, motor function z-score below −1). Cohen’s *d* effect size: 0.2 small, 0.5 moderate, and *d* = 0.8 strong effect [51]

### Comparison of cerebellar volumes between patients and controls

**Fig. 1:**
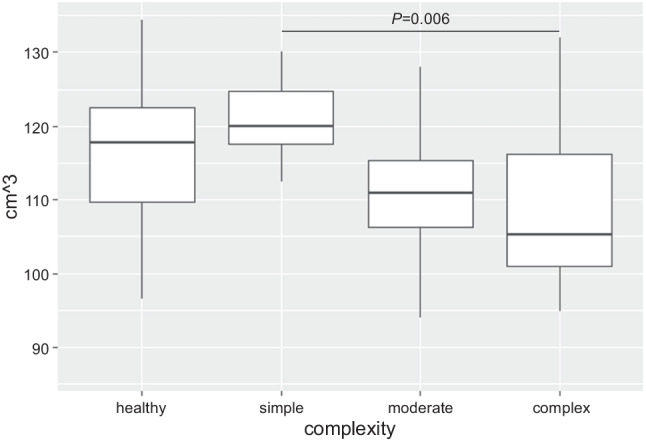
Posterior cerebellar lobe stratified by group and CHD complexity. In the patient group, more complex CHD was associated with a smaller posterior cerebellar lobe (*P =* 0.006 adjusted for total brain volume)

Total brain volume was smaller in patients compared to controls (B = −47.85, 95% CI: −90.8; −4.9, *β* = −0.22, *P =* 0.029); see Table 2. In contrast, total cerebellar volume was not different between patients and controls (B = 2.48, 95% CI: −2.1, 7.1, *β* = 0.088, *P =* 0.286, adjusted for total brain volume). When investigating cerebellar lobes separately, anterior cerebellar lobe and posterior cerebellar lobe were not different between patients and controls (anterior: B = −0.04, 95% CI: −0.6; 0.6, *β =* −0.01, *P =* 0.905; posterior: B = −1.52, 95% CI: −5.27, 2.24, *β* = −0.08, *P =* 0.425, adjusted for total brain volume). In the patient group, more complex CHD was associated with a smaller posterior cerebellar lobe (B = −4.3, 95% CI: −7.3, −1.2, *β* = −0.29, *P =* 0.006, overall model *P* < 0.001, adjusted for total brain volume); see Fig. 1. Similarly, there was a trend toward smaller anterior cerebellar lobe; however, the difference was not significant (B = −0.51, 95% CI: −1.05, 0.02, *β* = −0.19, *P =* 0.060, adjusted for total brain volume). There was no difference in total cerebellar volume depending on CHD complexity (B = −1.79, 95% CI: −6.5, 3.0, *β* = −0.08, *P =* 0.455). After adjusting for multiple comparisons, the posterior cerebellar lobe remained smaller in more complex CHD (*P* of the model <0.5).

**Table 2:** Brain volumetric measures in the cohort

M (SD)	ACHD, *N* = 43	Controls, *N* = 53	*P*
Total brain volume	1065.19 (114.03)	1113.04 (97.88)	0.029
Total cerebellar volume	136.42 (15.25)	138.03 (13.23)	0.286*
Posterior cerebellar lobe	113.62 (10.49)	116.69 (8.75)	0.425*
Anterior cerebellar lobe	16.50 (1.88)	16.85 (1.53)	0.905*

*M*, mean; *SD*, standard deviation

*Adjusted for total brain volume; brain volumes are given in cm^3^

### Association of cerebellar lobes with cognitive and motor function

In Table 3, the association of cognitive, executive, and motor functions with the anterior or posterior cerebellar lobe is illustrated. In the combined group, the posterior cerebellar lobe was not associated with the IQ, attention, and processing speed score (*P* > 0.1). Smaller posterior cerebellar lobe was associated with worse inhibitory control, but not after adjusting for multiple comparisons (*P* of the model = 0.06). Similarly, the anterior cerebellar lobe was not associated with pure motor functions, and adaptive motor functions were measured using the pegboard task (*P* > 0.1). When exploring the group of patients with moderate and complex CHD separately in post hoc analyses, we did not find a significant association of EF and motor functions with cerebellar lobes (*P* > 0.05).

**Table 3:** Association of cerebellar lobes with cognitive and motor function

	Posterior cerebellar volume			
	β	B	95% CI	*P*
IQ	0.06	0.07	−0.20; 0.35	0.596
Working memory	0.06	0.04	−0.10; 0.18	0.584*
Inhibition	−0.21	−0.13	−0.26; −0.00	0.047*
Flexibility	−0.06	−0.04	−0.17; 0.09	0.580
Planning	−0.05	−0.04	−0.24; 0.16	0.677
Fluency	−0.17	−0.11	−0.24;0.03	0.118
Attention	0.09	0.07	−0.09; 0.22	0.402
Processing speed	0.09	0.06	−0.08; 0.20	0.424
	Anterior cerebellar volume			
	β	B	95% CI	*P*
Pure motor function	0.11	0.08	−0.07; 0.25	0.308
Adaptive motor function	0.01	0.01	−0.13; 0.14	0.937

Linear regression analyses (dependent variable: functional outcome; independent variable: cerebellar volume; adjusted for total brain volume). *P* of the effect is reported

*Overall *P* of the model was significant, *P* < 0.05

## Discussion

In this current cohort of adults with CHD aged 18 to 32, we compared cerebellar volumes between patients and controls and explored the association of cerebellar volumes with executive and motor functions. Cerebellar volumes were similar between patients and controls after correcting for differences in total brain volume. Only the posterior cerebellar lobe was smaller in CHD compared to controls, in particular in patients with more complex CHD, and was associated with worse inhibitory control. We were not able to find an association of cerebellar volume with other EF, nor an association with motor functions.

General cognitive abilities and pure motor functions were significantly lower in ACHD compared to controls. However, ACHD performed within the normal range. Similar results have been previously described in numerous studies in CHD children, indicating only mild impairments in the CHD population present across several neurodevelopmental domains [52, 53], and increasing evidence suggests the persistence of EF and motor impairments in adults with CHD [28]. Of note, in our sample of adults with CHD, repetitive/alternating tapping movements were impaired, while there was no difference in the pegboard task compared to controls. This is in contrast to results from another study, reporting worse performance on the pegboard task in CHD adults compared to controls [11]. Differences in results may partially be attributed to differences in the patient population. The participants in the study by Semmel et al. had more severe CHD, with nearly half participants having a univentricular CHD diagnosis; all subjects underwent cardio-pulmonary bypass surgery, and prematurely born subjects >34 weeks of gestation were included. Consistent with the notion that motor impairments might be linked to smaller cerebellar volumes in severe CHD but not in more mild cases, another study found impaired psychomotor speed in adults with severe CHD, but not in the moderate CHD group [54]. Therefore, there is increasing evidence of persisting motor impairment beyond childhood in the CHD population, and healthcare professionals should consider the risk of motor dysfunction when counseling CHD adults.

In our cohort, total cerebellar volume and anterior cerebellar gray matter were similar in CHD patients and controls. However, in the patient group, the posterior cerebellar gray matter was smaller with increasing CHD complexity. This is in contrast to another study in CHD adults, where both anterior and posterior cerebellar gray matter volumes were reported to be smaller, although there were no differences in intracranial volume compared to healthy controls. However, in this previous study, total brain volume was not reported, and intracranial volume may poorly reflect total brain volume, as cerebrospinal fluid space may be enlarged in CHD patients with univentricular diagnosis [55]. Therefore, it is not known whether the cerebellar volume reductions reflect impaired global brain growth or whether cerebellar structures were particularly affected beyond the extent of total brain volume reductions. Of note, brain volumetric measures may be more affected by increasing CHD severity since posterior cerebellar volume reductions were more pronounced in our patients with complex CHD. Similarly, smaller brain volumes have been reported in adults with severe CHD, while brain volumes appear to be similar in adults with simple CHD and controls [56, 57]. Thus, patients with complex CHD may be particularly at risk for brain volume reductions.

We found an association of smaller posterior gray matter with worse inhibitory control, although this association did not survive FDR correction. This is consistent with another study in CHD adults and suggests that the cerebellum may contribute to inhibitory impairments in CHD adults [11]. We were not able to find an association between anterior or posterior cerebellar volumes and IQ, attention or processing speed, and most EF. This result stands in contrast to the association between posterior cerebellar volumes and executive functioning reported by Semmel et al. [11], although, consistent with the Semmel et al. study, we observed that anterior cerebellar volume was not associated with motor function impairments. In the preterm population, an association of the cerebellum with motor impairments has been previously reported [24]. While the preterm and CHD population show similar risk for neurodevelopmental impairment [58], the cerebellum may be more vulnerable in the preterm population, where cerebellar injury is considered a common complication of preterm birth [25]. Furthermore, cerebellar involvement in executive and motor dysfunction is complex and likely varies among individuals [18]. Although we tried to account for spatial compartmentalization of the cerebellum by investigating anterior and posterior cerebellar gray matter separately, we were not able to account for more individualized differences. It has been previously suggested that a cerebro-cerebellar loop could mediate EF performance [17], and thus, cerebellar volumes may poorly reflect cerebellar function and more advanced imaging techniques, such as diffusion tensor imaging to extract brain tracts, which may be more sensitive to involvement of the cerebellum in EF. In addition, lab-based tasks to assess EF skills usually involve several brain regions; for example, the color-word-inference task activates brain regions in the frontal, parietal, and occipital regions, yet research often investigates associations of EF with single brain regions [59]. Furthermore, participants may use different strategies to solve the same task, thus involving different brain regions [60]. Recently, the concept of separable EF domains has been challenged, with EF likely involving partly overlapping cognitive functions [61]. Due to these challenges, we used a large test battery to comprehensively assess EF in our ACHD participants.

Our study has some limitations. We were not able to investigate the effect of prematurity on cerebellar volume since gestational age was not collected as part of this study. In addition, we were not able to investigate the effect of a specific cardiac diagnosis on EF as we included a variety of CHD, although by including a range of cardiac diagnoses, our sample was more representative of the CHD general population. Our comprehensive test battery may have resulted in selecting mostly high-functioning CHD adults (IQ 97.5). Thus, detecting differences in cognitive and executive functioning may have been more difficult due to only mild impairments. However, it is important to study the participants with mild CHD to better understand the mechanism of neurocognitive impairment in the general CHD population.

In conclusion, cerebellar volumes were mostly unimpaired in our CHD cohort. The only structure smaller in CHD was the posterior cerebellar lobe, in particular in patients with complex CHD. This finding indicates an increased risk of cerebellar abnormalities in patients with complex CHD. We found weak evidence for an association between cerebellar posterior gray matter and worse inhibitory control. Other EF did not correlate with the cerebellar volumes, suggesting minor or more complex involvement of the cerebellum in EF. Larger studies with more advanced imaging techniques are necessary to further clarify the involvement of the cerebellum in EF.

### Supplementary information

ESM 1 (DOCX 13 kb)

## Data Availability

The de-identified data are available from the corresponding author upon reasonable request.

## Abbreviations

ACHD: adults with congenital heart disease
CHD: congenital heart disease
CI: confidence interval
EF: executive function
IQ: intelligence quotient
IQR: interquartile range
MRI: magnetic resonance imaging
SD: standard deviation
SE: standard error

## Declarations

### Reporting guidelines checklist

The STROBE checklist has been applied.
