# Supplementary material for: Association of cerebellar volume with cognitive and motor function in adults with congenital heart disease
Source: Neurol Sci. 2023 Jun 23;44(11):3979–87. doi: 10.1007/s10072-023-06861-2 (PMC10570150; doi:10.1007/s10072-023-06861-2)
Supplement: Supplementary file 1 — (DOCX 13 kb) [file 10072_2023_6861_MOESM1_ESM.docx]

Supplementary Table 1

| Working memory |  |  |
| --- | --- | --- |
| WAIS | Verbal digit span | Average of longest span forward and backward |
| WMS-R | Visual block span | Average of longest span forward and backward |
| Inhibition |  |  |
| CWI | Inhibitory control  (Condition 3 minus condition 1) | Completion time |
| SST | Response inhibition | Total score |
| Flexibility |  |  |
| CWI | Flexibility  (Condition 4 minus condition 3) | Completion time |
| TMT | Numbers and letters (Condition B) | Completion time |
| Planning |  |  |
| SLP | Constructive solution behavior | Total score |
| ROCF | Visuomotor planning (Copy trial) | Total score |
| Fluency |  |  |
| 5-Point Test | Nonverbal fluency | Correct figures |
| RWT | Verbal fluency (S-words) | Correct words |
| Attention |  |  |
| TAP | Auditory response | Mean reaction time |
| TAP | Visual response | Mean reaction time |
| Processing speed |  |  |
| CWI | Color naming (Condition 1) | Completion time |
| TMT | Numbers (Condition A) | Completion time |

WAIS: Wechsler Adult intelligence Scale; WMS-R: Wechlser Memory Scale; CWI: Color word interference test; SST: Stop signal task; TMT: Trail making test; SLP: Standardized Link’s Probe; ROCF: Rey Osterrieth Complex Figure Test; RWT: Regensburger Wortflüssigkeitstest; TAP: Test of attentional performance

Supplemental Table 2: Cardiac diagnosis stratified by simple, moderate and complex CHD:

|  | N (%) |
| --- | --- |
| Simple CHD | 15 (34.9) |
| Isolated congenital aortic valve disease | 6 (13.9) |
| Ventricular septal defect - repaired | 2 (4.6) |
| Isolated congenital mitral valve disease | 2 (4.6) |
| Previously ligated or occluded ductus arteriosus | 1 (2.3) |
| Repaired ventricular septal defect with tricuspid valve disease | 1 (2.3) |
| Mild pulmonary stenosis | 1 (2.3) |
| Small atrial septal defect | 1 (2.3) |
| Congenital mitral valve disease and small atrial septal defect | 1 (2.3) |
| Moderate CHD | 20 (46.5) |
| Coarctation of the aorta | 6 (13.9) |
| Tetralogy of Fallot | 3 (6.9) |
| Ventricular septal defect - with coarctation of the aorta | 3 (6.9) |
| Ebstein anomaly | 2 (4.6) |
| Ventricular septal defect - with right ventricular outflow tract obstruction | 2 (4.6) |
| Ventricular septal defect - with mitral disease | 2 (4.6) |
| Abnormal origin of the left pulmonary artery and ventricular septal defect | 1 (2.3) |
| Subvalvar aortic stenosis | 1 (2.3) |
| Complex CHD | 8 (18.6) |
| Transposition of the great arteries | 6 (13.9) |
| Fontan procedure | 2 (4.6) |
